# Telerreabilitação Cardiovascular: Uma Alternativa para Maior Disponibilidade da Reabilitação Cardiovascular e Metabólica no Brasil

**DOI:** 10.36660/abc.20240570

**Published:** 2025-03-27

**Authors:** Artur Haddad Herdy, Ariella Sebastião Mangia, Magnus Benetti

**Affiliations:** 1 Instituto de Cardiologia de Santa Catarina São José SC Brasil Instituto de Cardiologia de Santa Catarina, São José, SC – Brasil; 2 Núcleo de Cardio-oncologia e Medicina do Exercício Centro de Ciências da Saúde e do Esporte Universidade do Estado de Santa Catarina Florianópolis SC Brasil Núcleo de Cardio-oncologia e Medicina do Exercício, Centro de Ciências da Saúde e do Esporte (CEFID), Universidade do Estado de Santa Catarina (UDESC), Florianópolis, SC – Brasil

**Keywords:** Telerreabilitação, Telemonitoramento, Reabilitação Cardíaca, Doenças Cardiovasculares, COVID-19

## Abstract

**Figura Central:**
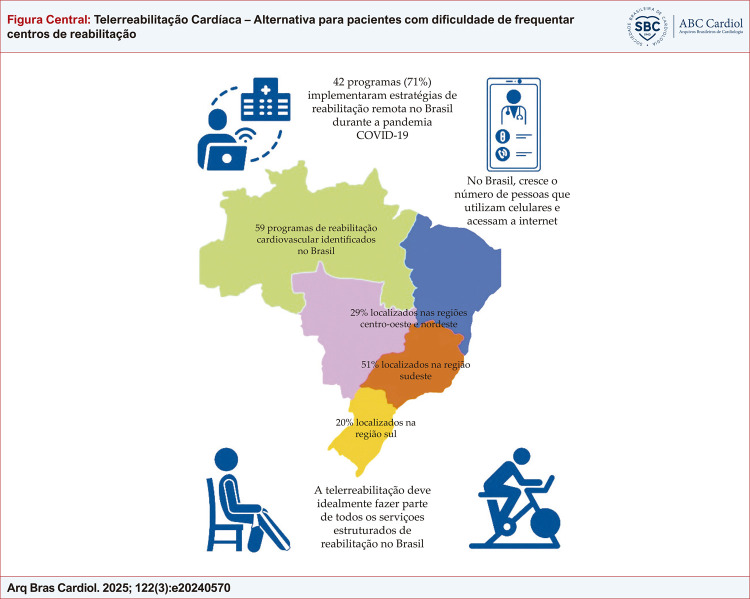
Telerreabilitação Cardíaca – Alternativa para pacientes com dificuldade de frequentar centros de reabilitação

A reabilitação cardiovascular (RCV) é vital para a prevenção de doenças cardiovasculares (DCV).^1 - 3^ Apesar de suas recomendações robustas, ainda é subutilizada globalmente devido ao baixo referenciamento, barreiras geográficas, como a falta de centros de RCV nos centros urbanos e falta de cobertura em áreas remotas, logística, além de limitações nos sistemas de saúde e na formação profissional.^4 , 5^ No Brasil, a situação é ainda mais desafiadora.^6^ A pandemia de COVID-19 expôs a fragilidade dos modelos tradicionais de RCV, impulsionando soluções inovadoras que assegurassem a continuidade dos cuidados. Nesse contexto, a telerreabilitação cardiovascular (TRC) surge como uma abordagem viável e acessível, representando uma solução moderna, alinhada às necessidades tecnológicas e culturais, devendo fazer parte idealmente de todos os programas de RCV no Brasil.

Até 2022, eram apenas 59 programas de RCV formais, sendo a maioria destes concentrados na região Sul e Sudeste do país.^7^ No ano seguinte, dados de um estudo que descreveu as características dos Programas de Reabilitação Cardíaca (PRCs) no Brasil e os impactos da COVID-19 mencionaram que dos 75 PRCs existentes,^8^ 59 participaram do estudo e 29% estavam localizados nas regiões centro-oeste e nordeste, 51% estavam localizados na região sudeste e 20% estavam na região sul, sendo que 42 PRCs (71%) implementaram estratégias de reabilitação remota durante a pandemia. Esses dados demonstram a escassez e a grande lacuna de vagas e serviços nas demais regiões brasileiras ( Figura Central ).

A RCV continua a ser estruturada da mesma forma que tem sido nos últimos anos, demonstrando incompatibilidade com o momento atual,^9^ tornando-se mais evidente após pandemia de COVID-19. A crise da COVID-19 trouxe desafios significativos para a entrega de serviços em reabilitação, destacando a urgência em encontrar soluções inovadoras e remotas que fossem capazes de assegurar a continuidade dos cuidados essenciais aos pacientes durante aquele momento.^10^ Nesse contexto, a TRC emergiu como uma solução para preencher essa lacuna.

## Telerreabilitação cardiovascular – conceito

A TRC é um programa que compreende um ou mais componentes da RCV tradicional, comumente ofertados em programas de RCV domiciliar, ou fora do ambiente hospitalar/centro especializado;^11^ é entregue por meio de tecnologia digital (telemonitoramento, e-learning, telecoaching).^12^

Tem por objetivo o manejo da DCV e a melhora de diferentes aspectos físicos, emocionais, psicológicos e sociais dos pacientes, em especial aqueles localizados em áreas com baixos níveis de acesso, seja pelo local de moradia ou dificuldade de acesso aos cuidados avançados.^4 , 13^

Dentre as diferentes formas de aplicação da TRC, as principais são aplicativos baseados em dispositivos móveis, *wearables* (vestíveis ou portáteis) e plataformas de gerenciamento de mídia social,^14^ que, utilizados em conjunto, são capazes de avaliar remotamente, realizar prescrições eletrônicas, ofertar educação em saúde e facilitar a comunicação entre médico, profissionais de saúde e paciente.^15^ Esta possibilidade de monitoramento de sinais vitais e supervisão próxima dos pacientes garantem a viabilidade da telemedicina e, portanto, da TRC, tornando-a um sistema alternativo à RCV tradicional.

O modelo da TRC, inclui os seguintes componentes:

1. Plano de tratamento individualizado: por meio de um programa abrangente que inclui exercício físico personalizado, aconselhamento de atividade física, orientação educacional sobre modificação dos fatores de riscos (cessação do tabagismo, gestão do diabetes, gestão da hipertensão e dislipidemia), e orientações nutricionais e psicológicas.
2. Utilização de algum sistema de telemonitoramento: permite a avaliação dos sinais vitais antes, durante ou após as sessões de exercício, podendo variar de acordo com os recursos e necessidades. Exemplos de dispositivos utilizados: monitoramento de eletrocardiograma (ECG) sem fio, monitores de frequência cardíaca (FC), monitores de pressão arterial (PA), monitores de saturação de oxigênio, pedômetros, e acelerômetros que utilizam dados geoespaciais e podem ser carregados via Bluetooth e smarthphone.

A entrega de um programa de TRC pode ocorrer das seguintes formas:^16^

- Virtual síncrono: interação audiovisual em tempo real entre paciente e equipe de RCV. Esse modelo pode beneficiar pacientes mais fragilizados que tenham menor capacidade funcional e necessitam de mais orientação e cuidado durante a prática de exercícios, além de favorecer as comunicações interpessoais e afetivas. Um ponto negativo é que as intervenções carecem de disponibilidade de tempo compatíveis entre equipe e paciente, além do número ideal de pacientes em um ambiente virtual simultaneamente ser desconhecido.
- Remoto assíncrono: Não há interação em tempo real entre paciente e equipe de RCV. O paciente recebe orientações de exercícios e pode relatar suas atividades físicas e outros dados de saúde por meio de tecnologia remota. Os benefícios desse modelo é a não restrição da quantidade de pessoas que podem ser contempladas pelas orientações de exercícios ou educacionais, por meio de videoaulas para executarem de acordo com a disponibilidade pessoal.
- Híbrida: alguns componentes da RCV são entregues presencialmente com o paciente e equipe no mesmo local e outras sessões são combinadas síncronas ou assíncronas remotas, apara atender melhor às necessidades do paciente.

A forma como a troca de dados ocorre na TRC classificada como síncrona beneficia o paciente na motivação e segurança da intervenção proposta, pois permite verificar as condições gerais dos participantes, ajustar a intensidade do exercício e fornecer instruções e feedback imediato.^17^

### Evidências em telerreabilitação

As investigações e análises da TRC têm sido objeto de crescente interesse em diversas nações, sobretudo após a pandemia de COVID-19, que acarretou uma significativa transformação no panorama da RCV.

Os programas de telessaúde em RCV existem desde o início da década de 1980, ofertados em áreas rurais, onde as barreiras geográficas dificultavam a participação em um programa tradicional de RCV.^10^

Em 2014, um estudo pioneiro em TRC avaliou o impacto de um modelo de cuidados domiciliares baseado em smartphones e comparou com um programa de RCV tradicional em pacientes após infarto do miocárdio. Os principais resultados foram maior adesão e taxa de conclusão ao programa baseado em smartphones comparado ao tradicional (94% vs. 68% e 80% vs. 47%, respectivamente). Para os desfechos clínicos e qualidade de vida, ambos os grupos obtiveram melhorias significativas no teste de caminhada de 6 minutos e na ingestão alimentar. Reduções significativas no peso e circunferência abdominal e melhores resultados no estado emocional e na qualidade de vida relacionada à saúde foram superiores no modelo domiciliar baseado em smartphones, além de uma leve redução na pressão diastólica final de 6 semanas também ter sido observado neste grupo. Este estudo acompanhou os pacientes por um período de 6 meses e observou que as melhorias observadas ao final do programa (6 semanas), especialmente o teste de caminhada e as melhorias psicossociais, foram mantidas a longo prazo.^18^

Em 2018, a Associação Holandesa de Cardiologia publicou um adendo à diretriz multidisciplinar de reabilitação cardíaca específica sobre este “novo” modelo de TRC, sendo um dos primeiros no mundo a estabelecer condutas profissionais na área.^19 , 20^

Grandes estudos de revisão sistemática e meta-análises foram realizados em países da Ásia Oriental, publicados nos últimos dois anos, com fortes evidências sobre eficácia e segurança,^21^ efetividade de longo prazo^15^e comparação entre TRC e RCV tradicional.^22^ Diferentes grupos de pacientes foram estudados, desde os menos complexos, como pós-intervenção coronária,^23^ até cenários mais desafiadores, como em pacientes com insuficiência cardíaca nos quais a TRC demonstrou ser viável e segura.^24^

A TRC ganhou mais evidência no período da pandemia de COVID-19, momento em que os maiores centros de reabilitação cardíaca tradicional fecharam (momentaneamente ou a longo prazo)^10 , 25 , 26^ e muitos profissionais de saúde optaram pela abordagem “online”, explorando as diversas aplicações móveis como os smartphones e smartwatches.^27^ No Brasil, um estudo transversal investigou os impactos da primeira onda epidemiológica da COVID-19 sobre os programas de RCV e verificou que somente dois programas, dos 75 existentes no território brasileiro, utilizaram plataformas digitais durante a pandemia de COVID-19.^28^ O mesmo estudo constatou também que, para manter os participantes nos programas de RCV, as atividades remotas por meio de chamadas por vídeo (33%) foram a estratégia mais utilizada, seguidas de utilização de fotos e vídeos (27%).

Uma síntese dos resumos das publicações pode ser visualizada no suplemento, tabela 1 .

### Proposta de desenvolvimento e implementação da telerreabilitação no Brasil

Na atualidade, desconhecemos quais programas de RCV do Brasil, em especial na rede pública, têm abordado a telerreabilitação como forma de atendimento para aqueles pacientes que são impedidos de participar de programas de RCV em decorrência de barreiras geográficas e de acesso.

Embora evidências demonstrem os benefícios clínicos e econômicos da TRC,^29 , 30^ infelizmente, as intervenções ficam limitadas a ambientes de pesquisa e raramente são implementadas aos cuidados regulares, o que pode ocorrer, em parte, devido ao desconhecimento da TRC e dos desafios de sua implementação.

A implementação bem-sucedida de programas de TRC requer uma aliança entre os principais centros de pesquisa e os grandes centros de RCV em nível nacional. A colaboração entre essas instituições pode possibilitar a realização conjunta de estudos para testar protocolos eficazes, garantindo a efetividade desse método inovador.

Um recente estudo destaca os desafios relacionados ao uso da tecnologia, especialmente entre os usuários mais idosos, como uma dificuldade de implementação da TRC, sugerindo a necessidade de revisar os dispositivos digitais disponíveis para pacientes idosos com doença coronariana. Os autores também destacam a necessidade de promover a TRC por meio de políticas e gestão de saúde, a fim de reduzir as desigualdades, assim como projetar intervenções que promovam a adesão a longo prazo e impacte em mudança no estilo de vida a longo prazo.^12^

Nesse contexto, investimentos substanciais em infraestrutura tecnológica são essenciais, tanto por parte de instituições públicas quanto privadas para que a adoção de programas de TRC ocorram no Brasil, via Sistema Único de Saúde e redes privadas.

As parcerias entre Universidades e centros de RCV podem ser um caminho inicial para implementar, validar e expandir esse modelo de entrega de RC para a realidade do Brasil. Embora a dificuldade em educação digital mencionada seja conhecida, acredita-se que as maiores barreiras para a implementação da TRC no Brasil seja a preparação e capacitação das equipes de profissionais que irão lidar com a tecnologia. É essencial que esses profissionais estejam devidamente treinados e capacitados para instruir e orientar os pacientes, de forma que compreendam como utilizar a tecnologia para realizar seus tratamentos por meio da telemedicina. No Brasil, observa-se um crescimento contínuo no número de pessoas que utilizam celulares e acessam a internet. Em 2023, 213 milhões de pessoas possuíam assinatura de celular móvel e 84% da população utilizavam a internet.^31^ Além disso, há uma variedade de plataformas de mídias sociais, serviços de streaming e aplicativos de treinamento físico, que oferecem possibilidades adaptadas a usuários com diferentes perfis de competência digital. Por outro lado, mesmo que pacientes mais idosos enfrentem maiores dificuldades em utilizar a tecnologia, eles podem contar com o apoio de familiares e cuidadores, desde que estes também recebam a devida orientação. O suporte dos familiares ou cuidadores torna-se essencial para viabilizar o tratamento de forma eficaz.

### Triagem e avaliações

A triagem dos pacientes elegíveis à TRC deve, idealmente, ser realizada por um médico cardiologista ou, na ausência deste, pelo médico responsável pelo programa. É recomendado, mas não impeditivo, que pacientes realizem o teste ergométrico (TE) e/ou teste cardiopulmonar de exercício (TCPE) antes de iniciarem seu programa de exercícios para identificação da zona alvo de treinamento, assim como é recomendado em programas de RCV tradicionais.^32 , 33^Na impossibilidade da realização do TE ou TCPE, o Talk Test^34^ ou teste de caminhada de 6 minutos^35^ podem auxiliar a identificar a intensidade do exercício adequada para melhorar a aptidão cardiorrespiratória.^16^

O profissional responsável pela prescrição e acompanhamento das sessões de exercício, seja o profissional de educação física ou fisioterapeuta, deve realizar uma anamnese detalhada, além de testes de avaliação da capacidade funcional e antropometria, para complementar as informações sobre estado geral do paciente, prévio aos exercícios. Reavaliações após a intervenção são recomendadas, principalmente para propiciar ao paciente informações sobre o seu progresso no programa. Desta forma, acredita-se que a motivação e as chances de mudança no estilo de vida do paciente, possam ser favorecidas.

Pacientes que apresentarem arritmia ventricular e/ou isquemia miocárdica sintomática importante durante o teste de esforço são considerados de maior risco ao treinamento físico supervisionado remotamente. Nesses casos, os riscos e benefícios devem ser avaliados individualmente.^19^

Ainda quanto à elegibilidade e segurança do paciente, atenção especial deve ser dada aos pacientes com insuficiência cardíaca grave, fragilidade, deficiência ortopédica ou cognitiva, que vivem sozinhos ou que se sentem desconfortáveis com o uso da tecnologia.^10^

Um protocolo de contingência em caso de evento adverso grave, como parada cardiorrespiratória, ou mesmo eventos adversos moderados a leves, deve estar descrito no programa de TRC e deve ser repassado ao paciente, familiares e cuidadores. Recomenda-se incluir os seguintes procedimentos:

1. Contatos de emergência: fornecer aos pacientes os números de contato do Serviço de Atendimento Móvel de Urgência (SAMU) e do Corpo de Bombeiros. Esses números devem estar acessíveis e visíveis, facilitando o acionamento em emergências.
2. Orientação para comunicação eficiente: instruir os pacientes sobre a forma adequada de comunicação com os atendentes de emergência:
  - Quem ligar, deverá informar aos atendentes que o caso envolve portadores de cardiopatia, especificando o tipo de cardiopatia diagnosticada;
  - Quem ligar, deverá informar aos atendentes que o caso envolve alguém que realiza exercícios supervisionados como parte de um programa de reabilitação;
  - Os sintomas apresentados, como dor no peito, falta de ar, tontura, entre outros, devem ser descritos com precisão.
  - O endereço e o local de acesso em que o paciente receberá o atendimento deverá ser informado.
3. Capacitação dos cuidadores: disponibilizar aos cuidadores uma videoaula abrangente sobre reanimação cardiopulmonar (RCP), com o objetivo de prepará-los para eventuais situações de parada cardiorrespiratória. Alternativamente, quando possível, essas orientações podem ser fornecidas por meio de palestra presencial, em modelo híbrido.

Este plano visa garantir que os pacientes e cuidadores estejam adequadamente preparados para responder de forma eficaz em situações emergenciais, promovendo maior segurança durante a prática de telerreabilitação.

### Prescrição das sessões de exercício físico na telerreabilitação

O conteúdo de um programa de exercícios supervisionados remotamente deve ser personalizado, com base nos objetivos, preferências e habilidades funcionais individuais de cada paciente, de maneira similar a um programa de RCV tradicional. A experiência e vivência prévias do paciente na modalidade escolhida podem aumentar a adesão, motivação e a segurança. Os modelos de RCV híbrida, que combinam sessões de exercícios em centros especializados com TRC, têm sido alvo de pesquisas e tem ganhado popularidade com evidências em desenvolvimento.^4 , 36^

O treinamento físico deve ser prescrito de acordo com o modelo FITT (frequência, intensidade, tempo/duração e tipo de exercício).^1 , 37^ A frequência dos exercícios aeróbios deve ser de pelo menos três dias por semana, preferencialmente todos os dias. Intercalar o exercício físico com atividades físicas realizadas no domínio do lazer ou de deslocamento pode ser uma boa estratégia para aumentar o nível de atividade física. Para os exercícios resistidos, recomenda-se uma frequência de pelo menos duas vezes por semana, com ênfase em atividades que melhorem as habilidades necessárias para as atividades de vida diária, de forma eficiente e segura, com foco em movimentos naturais e funcionais, como empurrar, puxar, agachar, levantar e girar. Podem ser utilizados pesos livres, elásticos, cadeira, bastão, bola, over ball, entre outros acessórios.

Para a prescrição da intensidade, recomenda-se orientação individualizada, combinando diferentes variáveis obtidas pelo TCPE ou TE com score individual de percepção subjetiva do esforço (Borg) ou teste de fala. A recomendação básica é considerar um domínio de intensidade moderado ou moderado a alto, quando possível (pacientes de baixo risco), ajustado às características individuais do paciente.^1^ Em pacientes que realizam o TCPE, pode-se utilizar como referência a frequência cardíaca observada nos limiares aeróbio e anaeróbio, iniciando próximo ao primeiro limiar e progredindo para FC entre os limiares 1 e 2. No TE, a intensidade pode variar de 60% a 80% da FC máxima alcançada no teste, evoluindo para até 90% da FC máxima. Também pode-se utilizar 50 a 70% da FC de reserva para pacientes sem teste. As sessões podem ter duração entre 30 e 60 minutos. Diferentes tipos de exercícios físicos podem ser realizados: exercícios resistido, de mobilidade, flexibilidade, com o peso corporal, caminhadas, e exercícios em ergômetros.

Destaca-se que, diante dos diferentes protocolos dos estudos realizados até o momento, não se conhece a intensidade ótima para a TRC. Observa-se que as recomendações seguem um padrão semelhante às descritas em programas de RCV baseada em centros tradicionais, o que viabiliza estudos adicionais e padronização de protocolos, para que possamos dispor de informações mais robustas. Surge a hipótese de que a adesão ao programa de exercícios físicos e a sua manutenção a longo prazo sejam mais impactantes em desfechos de saúde do que a intensidade dos exercícios realizados.

As sessões de exercício físico remotas devem ser prescritas e supervisionadas por profissionais de educação física e fisioterapeutas habilitados e capacitados, preferencialmente com experiência em programas de RCV em centros especializados. Recomenda-se que pacientes de fase II, antes de iniciarem um programa remoto, tenham a vivência mínima de 4 sessões presenciais em centro especializado. Essa abordagem híbrida tem como objetivo proporcionar uma prática de percepção aos esforços, orientar e corrigir movimentos, e direcionar a prática remota das sessões. Nesse momento, é aconselhável que o paciente receba orientações de uso da escala de percepção subjetiva do esforço,^38^ que deverá ser utilizada em conjunto com monitores de frequência cardíaca durante as sessões de exercício.

Ainda na fase II, as sessões de exercício online devem ocorrer em tempo real, com comunicação bilateral entre o profissional de saúde e o paciente. Recomenda-se que o paciente faça uso de algum monitor de sinais vitais, como frequencímetro ou oxímetro, que esteja na presença de um acompanhante e que tenha ao seu alcance contatos de emergência para casos de ocorrência de eventos adversos. Minimizar os riscos e priorizar a segurança são recomendações emergenciais.

Na fase III, as sessões podem ser assíncronas, por meio de videoaulas ou de orientação individualizada utilizando aplicativos específicos para a prescrição de exercícios.

Atualmente, existem aplicativos para prescrição de exercícios em academia de musculação ou com o peso corporal, que podem ser executados em casa ou academias de forma assíncrona. Esses novos aplicativos são simples de utilizar e permitem a visualização de vídeos explicativos, com informações sobre execução, número de séries, repetições e cargas. Esta tecnologia proporciona a entrega de programas de exercícios físicos personalizados ou em grupo, e ganhou destaque durante a pandemia de COVID-19, sendo cada vez mais utilizada em diferentes populações.

## Conclusão

Considerando o Brasil um país de dimensões continentais, a telerreabilitação cardíaca surge como uma solução relativamente simples e eficaz para proporcionar uma maior oferta de RCV, principalmente em áreas carentes de serviços disponíveis para a população. As plataformas digitais possibilitam treinamento universal para diversos serviços, com possibilidades de criação de centros coordenadores e consultores especializados. A telerreabilitação deve idealmente fazer parte de todos os serviços estruturados de RCV no Brasil.

## *Material suplementar

Para informação adicional, por favor, clique aqui


