# Supplementary material for: Telerreabilitação Cardiovascular: Uma Alternativa para Maior Disponibilidade da Reabilitação Cardiovascular e Metabólica no Brasil
Source: Arq Bras Cardiol. 2025 Mar 27;122(3):e20240570. [Article in Portuguese] doi: 10.36660/abc.20240570 (PMC12058155; doi:10.36660/abc.20240570)
Supplement: Supplementary file 1 [file 0066-782X-abc-122-3-e20240570-suppl01.pdf]

Suplemento

Tabela 1 - Resumo das características analisadas nos estudos de meta-análises

Autor, ano de publicação, país e revista	Nº de estudos incluídos, (n) amostral, Patologia Grupo experimental (GE)/ Grupo controle (GC)	Objetivo do estudo	Intervenção e Exercício Físico	Utilização de dispositivos vestíveis? Forma de entrega da telereabilitação	Desfecho	Resultados
(YANG; JIA; LI; MEI *et al.*, 2023)[1] China Occupational Therapy International A3	8 ECR (n= 1578) DCV (GE)= HCTR (GC) = CU	Eficácia e segurança na HCTR em comparação com cuidados usuais de reabilitação	Programa de exercícios físicos, educação sobre fatores de risco, suporte psicológico no estilo de vida entregues por tecnologia de comunicação	Sim, sensores vestíveis ou smartwatch	Taxas de readmissão e mortalidade; qualidade de vida (SF-36), (VO_2 pico) e (TC6	HCTR e cuidados usuais oferecem resultados semelhantes nas reduções das taxas de readmissão, mortalidade e na melhoria da QV. HCTR foi superior para melhorar o VO_2 pico e TC6. Alternativa segura e eficaz para DCV
(ZHONG; LIU; CHENG; XU *et al.*, 2023) [2] China JMIR Mhealth Uhealth A	10 ECR (n=1417) DAC (GE)= HCTR (GC) = CU	Avaliar efetividade a longo prazo da telereabilita-ção	Telerreabilitação cardíaca baseada em intervenção no exercício, o controle dos fatores de risco, avaliação médica, combinações dietéticas razoáveis e aconselhamento psicossocial; Intervenção de exercício, com intensidade predominante-mente moderada, frequência cardíaca de treinamento equivalente a 70%-80% da frequência cardíaca de reserva e controle individualizado por meio de monitorização eletrônica.	Sim, diferentes equipamentos de aplicação remota	Aptidão cardiopulmonar; fatores de risco cardiovascular (PA, IMC, lipídeos séricos), escalas psicológicas (ansiedade, depressão, QV); adesão a TRC e eventos adversos	Melhorou o VO_2 pico após 6 meses de TRC. Não houve redução no controle dos fatores de risco cardiovascular, nos scores de ansiedade ou depressão. Melhorou a QV a longo prazo. 80% de taxa de conclusão e baixa incidência de efeitos adversos a longo prazo

(ZHONG; FU; XU; SUN *et al.*, 2023) [3]<br><br>China<br><br>BMC Cardiovas cular Disorders – A3	5 ECR (n= NA)<br><br>ICP<br><br>(GE)= HBCTR<br><br>(GC) = RC ambulatorial, ou CU ou ambulatorial ativa	Investigar o efeito dos programas de telereabilita- ção cardíaca domiciliar em pacientes submetidos a (ICP)	Os programas de exercícios variaram entre os estudos, mas geralmente envolviam exercícios físicos supervisionados e monitorados em tempo real, alguns prescritos individualmente, como caminhada ao ar livre ou adaptados às necessidades individuais	Sim, telemóveis, tablets, computadores, televisão ou vídeo conferência	Função física (TC6) e QV;<br><br>PA, perfil lipídico completo, ansiedade e depressão	Melhorou a capacidade de exercício no teste de (TC6); não houve diferença significativa na QV; Reduziu a Pressão arterial sistólica, diastólica não alterou; melhorou os níveis de triglicerídeos e colesterol de lipoproteínas de baixa densidade; sem alterações significativas no colesterol total e ao colesterol de lipoproteína de alta intensidade
(JIN CHOO; CHANG, 2022) [4]<br><br>Coréia do Sul<br><br>Medicine	8 ECR (n=750)<br><br>DC<br><br>(GE)= TCR e RC em centro	Investigar a efetividade da telereabilita- ção cardíaca comparada com a reabilitação em centro	Telereabilita- ção: exercícios aeróbios com intensidade variando entre 70% a 80% da frequência cardíaca de reserva 40 min; 3x na semana, durante 12 semanas<br><br>RC em centros: exercícios aeróbios com intensidade de 70%a 85% da frequência cardíaca máxima 40 min; 3x na semana, durante 12 semanas	Monitor de FC, sensores vestíveis, sensores de movimento.<br><br>Foram utilizadas ligações telefônicas, envio de emails, SMS, arquivos de áudio e vídeo, aplicativo via web e smartphone	Aptidão cardiorrespiratória, pressão arterial, lipídios sanguíneos, composição corporal, qualidade de vida	Indicam semelhança entre telereabilitação cardíaca e reabilitação em centro na aptidão cardiorrespiratória, pressão arterial sistólica e diastólica, composição corporal e qualidade de vida. Resultados não significativos para colesterol total e qualidade de vida em termos de saúde mental
(RAMACHANDRAN; JIANG; TAM; YEO *et al.*, 2022) [5]<br><br>Singapura<br><br>European Journal of Preventive Cardiology<br><br>A1	14 ECR (n= 2869)<br><br>(DC) na fase II<br><br>(GE)= HBCTR<br><br>(GC) = CBCR ou CU sem nenhum treinamento físico supervisionado ou estruturado	Avaliar eficácia da TRC domiciliar de fase II em comparação com a RC convencional ou cuidados usuais.	Frequência semana de exercícios de 3 a 5 vezes por semana, duração de 30 min. Intensidade definida de acordo com FC no limiar anaeróbio.	Estudos que utilizaram qualquer aplicação de saúde móvel, plataforma baseadas na web, aplicativos de smartphone e dispositivos de telemonitora- mento da atividade física dos participantes.	Medidas de comportamen- tais, fisiológicas e clínicas: atividade física, pressão arterial, perfil lipídico, qualidade de vida, mortalidade, hospitalização, adesão à medicação, tabagismo e depressão; segurança, adesão e satisfação dos pacientes	A telereabilitação caradíaca domiciliar parece ser tão eficaz quanto a RC tradicional, e em alguns casos mais eficaz que os cuidados usuais, para melhorar a capacidade funcional, AF, QV e escores de depressão.

Autores: Herdy, A. H.; Mangia, A.S.; Benetti, M.

Legenda – CU:cuidados usuais; DAC: doença arterial coronariana; DC: doença coronariana; DCV: doença cardiovascular; ECR: ensaio clínico randomizado; FC: frequência cardíaca; HBCTR: telereabilitação cardíaca domiciliar; HCTR: telereabilitação cardíaca home-based; ICP: Intervenção coronária percutânea; IMC: índice de massa corporal; NA: não há; PA: pressão arterial; QV: qualidade de vida; SF-36Short Form-

36 Health Status Questionnaire; RC: reabilitação cardíaca; TRC: telereabilitação cardíaca; VO_2 pico: consumo de oxigênio de pico; TC6: teste de caminhada de 6 minutos.

Referências

1. Yang Z, Jia X, Li J, Mei Z, Yang L, Yan C, et al. Efficacy and Safety of Hybrid Comprehensive Telerehabilitation (HCTR) for Cardiac Rehabilitation in Patients with Cardiovascular Disease: A Systematic Review and Meta-Analysis of Randomized Controlled Trials. Occup Ther Int. 2023;2023:5147805.
2. Zhong W, Liu R, Cheng H, Xu L, Wang L, He C, et al. Longer-Term Effects of Cardiac Telerehabilitation on Patients With Coronary Artery Disease: Systematic Review and Meta-Analysis. JMIR Mhealth Uhealth. 2023;11:e46359.
3. Zhong W, Fu C, Xu L, Sun X, Wang S, He C, et al. Effects of home-based cardiac telerehabilitation programs in patients undergoing percutaneous coronary intervention: a systematic review and meta-analysis. BMC Cardiovasc Disord. 2023;23(1):101.
4. Jin Choo Y, Chang MC. Effects of telecardiac rehabilitation on coronary heart disease: A PRISMA-compliant systematic review and meta-analysis. Medicine (Baltimore). 2022;101(28):e29459.
5. Ramachandran HJ, Jiang Y, Tam WWS, Yeo TJ, Wang W. Effectiveness of home-based cardiac telerehabilitation as an alternative to Phase 2 cardiac rehabilitation of coronary heart disease: a systematic review and meta-analysis. Eur J Prev Cardiol. 2022;29(7):1017-43.
