# Supplementary material for: Telerreabilitação Cardiovascular: Uma Alternativa para Maior Disponibilidade da Reabilitação Cardiovascular e Metabólica no Brasil
Source: Arq Bras Cardiol. 2025 Mar 27;122(3):e20240570. [Article in Portuguese] doi: 10.36660/abc.20240570 (PMC12058155; doi:10.36660/abc.20240570)
Supplement: Translation

# Translation: Cardiovascular Telerehabilitation: An Alternative for Greater Availability of Cardiovascular and Metabolic Rehabilitation in Brazil

**DOI:** 10.36660/abc.20240570i

**Authors:** Artur Haddad Herdy, Ariella Sebastião Mangia, Magnus Benetti

**Affiliations:** 1 Brazil Instituto de Cardiologia de Santa Catarina, São José, SC – Brazil; 2 Brazil Núcleo de Cardio-oncologia e Medicina do Exercício, Centro de Ciências da Saúde e do Esporte (CEFID), Universidade do Estado de Santa Catarina (UDESC), Florianópolis, SC – Brazil

**Keywords:** Telerehabilitation, Telemonitoring, Cardiac Rehabilitation, Cardiovascular Diseases, COVID-19

## Abstract

**Central Illustration:** Cardiac Telerehabilitation – An alternative for patients with difficulty attending rehabilitation centers

Cardiovascular rehabilitation (CVR) is vital for the prevention of cardiovascular diseases (CVD).^1 - 3^Despite robust CVR recommendations, it is still underutilized globally due to low referral, geographical barriers, such as the lack of CVR centers in urban areas and the lack of coverage in remote areas, logistics, and limitations in healthcare systems and professional training.^4 , 5^In Brazil, the situation is even more challenging.^6^The COVID-19 pandemic exposed the fragility of traditional CVR models, driving innovative solutions to ensure the continuity of care. In this context, cardiovascular telerehabilitation (CTR) emerges as a viable and accessible approach, representing a modern solution that is aligned with technological and cultural needs and should ideally be part of every CVR program in Brazil.

By 2022, there were only 59 formal CVR programs, most of which were concentrated in the South and Southeast regions of Brazil.^7^The following year, data from a study that described the characteristics of Cardiac Rehabilitation Programs (CRPs) in Brazil and the impacts of COVID-19 showed that of the 75 existing CRPs,^8^59 participated in the study and 29% were located in the Mid-West and Northeast regions, 51% were located in the Southeast region, and 20% in the South region, with 42 CRPs (71%) implementing remote rehabilitation strategies during the pandemic. These data demonstrate the scarcity and the lack of vacancies and services in other Brazilian regions ( Central Illustration ).

The CVR has been structured without change over the last few years, and this has shown to be inconsistent with the current times,^9^becoming even more evident after the COVID-19 pandemic. The COVID-19 crisis has brought significant challenges to the delivery of rehabilitation services, highlighting the urgency for innovative and remote solutions that can ensure the continuity of essential care for patients during this time.^10^In this context, CTR has emerged as a solution to fill this gap.

## Cardiovascular telerehabilitation – concept

CTR is a program that comprises one or more components of traditional CVR, commonly offered in CVR programs at home or outside a hospital/specialized center environment;^11^it is delivered through digital technology (telemonitoring, e-learning, telecoaching).^12^

Its objective is to manage CVD and improve different physical, emotional, psychological, and social aspects of patients, especially those located in areas with low levels of access, whether due to their place of residence or difficulty in accessing advanced care.^4 , 13^

Among the different forms of application of CTR, the main ones are based on mobile devices, wearables, and social media management platforms,^14^which, when used together, can remotely evaluate, make electronic prescriptions, offer health education, and facilitate communication between physicians, healthcare professionals, and patients.^15^The possibility of closely monitoring vital signs and patients guarantees the viability of telemedicine and, therefore, of CTR, making it an alternative system to traditional CVR.

The CTR model includes the following components:

1. Individualized treatment plan: through a comprehensive program covering personalized physical exercise, physical activity counseling, guidance on modifying risk factors (smoking cessation, diabetes management, and hypertension and dyslipidemia management), and nutritional and psychological counseling.
2. Use of a telemonitoring system: allows the assessment of vital signs before, during, or after exercise sessions, and may vary according to resources and needs. Examples of devices used are wireless electrocardiogram (ECG) monitoring, heart rate (HR) monitors, blood pressure (BP) monitors, oxygen saturation monitors, pedometers, and accelerometers that use geospatial data and can be downloaded via Bluetooth and a smartphone.

The delivery of a CTR program can occur in the following ways:^16^

- Synchronous Virtual: real-time audiovisual interaction between the patient and the CVR team. This model can be beneficial to more fragile patients with reduced functional capacity, who require more guidance and care during exercise. This model also fosters interpersonal and emotional communication. A negative point is that interventions lack compatible time availability between the team and the patient, in addition to the ideal number of patients simultaneously in a virtual environment being unknown.
- Asynchronous Remote: There is no real-time interaction between the patient and the CVR team. The patient receives exercise guidance and can report their physical activities and other health data through remote technology. The benefits of this model are that there is no restriction on the number of people who can receive exercise or educational guidance through video classes, who then can perform the exercise according to their personal availability.
- Hybrid: Some components of CVR are delivered in person with the patient and team in the same location, and other sessions are combined synchronously or asynchronously remotely to meet the patient’s needs best.

The way in which data exchange occurs in synchronous CTR benefits the patient in terms of motivation and safety of the proposed intervention. In this model, the team can check the general conditions of the participants, adjust the intensity of the exercise, and provide instructions and immediate feedback.^17^

### Evidence in Telerehabilitation

CTR investigations and analyses have been the subject of growing interest in several nations, especially after the COVID-19 pandemic, which brought about a significant transformation in the CVR landscape.

CVR telehealth programs have existed since the early 1980s, offered in rural areas where geographic barriers made participation in a traditional CVR program difficult.^10^

In 2014, a pioneering CTR study evaluated the impact of a smartphone-based home care model and compared it with a traditional CVR program in patients after myocardial infarction. The main results were higher adherence and completion rates for the smartphone-based program compared to the traditional one (94% vs 68% and 80% vs 47%, respectively). As for clinical outcomes and quality of life, both groups achieved significant improvements in the 6-minute walk test and food intake. The smartphone-based home group showed significant reductions in weight and waist circumference, and better improved emotional state and health-related quality of life outcomes. It also showed a slight reduction in 6-week end-diastolic blood pressure. This study followed patients for 6 months and observed that the improvements seen at the end of the program (6 weeks), especially the walking test and psychosocial improvements, were maintained in the long term.^18^

In 2018, the Dutch Association of Cardiology published an addendum to the multidisciplinary cardiac rehabilitation guideline specifically covering this “new” CTR model, being one of the first in the world to establish professional guidelines in the area.^19 , 20^

Large systematic review studies and meta-analyses have been conducted in East Asian countries, published in the last two years, with strong evidence on efficacy and safety,^21^long-term effectiveness,^15^and comparison between CTR and traditional CVR.^22^Different groups of patients were studied, from the least complex, such as post-coronary intervention,^23^to more challenging scenarios, such as patients with heart failure in whom CTR proved to be viable and safe.^24^

CTR gained more prominence during the COVID-19 pandemic when the largest traditional cardiac rehabilitation centers closed (temporarily or long-term),^10 , 25 , 26^and many healthcare professionals resorted to the “online” approach, exploring various mobile applications such as smartphones and smartwatches.^27^In Brazil, a cross-sectional study investigated the impacts of the first epidemiological wave of COVID-19 on CTR programs and found that only two programs, out of 75 existing in Brazil, used digital platforms during the COVID-19 pandemic.^28^The same study also found that to maintain participants in CTR programs, the most used strategy was remote activities through video calls (33%), followed by the use of photos and videos (27%).

A summary of the publications’ abstracts can be seen in the supplement, Table 1 .

### Proposal for the development and implementation of telerehabilitation in Brazil

Currently, we do not know which CVR programs in Brazil, especially in the public network, have addressed telerehabilitation as a form of care for those patients who are prevented from participating in CVR programs due to geographical and access barriers.

Although evidence demonstrates the clinical and economic benefits of CTR,^29 , 30^unfortunately, interventions are limited to research settings. They are rarely implemented in regular care, which may be in part due to a lack of awareness of CTR and the challenges of its implementation.

Successful implementation of CTR programs requires an alliance between leading research centers and major CVR centers at the national level. Collaboration between these institutions can enable joint studies to test effective protocols, ensuring the effectiveness of this innovative method.

A recent study highlights the challenges associated with the use of technology, especially among elderly users, as a barrier to implementing CTR, suggesting the need to review the digital devices available for older patients with coronary heart disease. The authors also highlight the need to promote CTR through healthcare policies and health management to reduce inequalities, as well as to design interventions that promote long-term adherence and impact long-term lifestyle change.^12^

In this context, substantial investments in technological infrastructure are key, both by public and private institutions, so that CTR programs are implemented in Brazil through the country’s Unified Health System and private networks.

Partnerships between Universities and CVR centers can be a way to implement, validate, and expand the CR delivery model according to the reality of Brazil. In addition to the issue of digital education, it is believed that the greatest barriers to the implementation of CTR in Brazil are the qualification and training of the professionals who will deal with the technology. These professionals must be properly trained and qualified to instruct and guide patients so that they understand how to use technology to carry out their treatments through telemedicine. In Brazil, the number of people using smartphones and accessing the Internet is growing. In 2023, 213 million people had a mobile phone subscription and 84% of the population used the Internet.^31^In addition, there is a variety of social media platforms, streaming services, and fitness apps that offer different possibilities to users according to their different digital competence profiles. On the other hand, when elderly patients face problems using technology, they can count on the support of family members and caregivers, as long as they also receive appropriate guidance. The support of family members or caregivers is key to making treatment effective.

### Screening and Assessments

Screening of patients eligible for CTR should ideally be performed by a cardiologist or, when a cardiologist is not available, by the physician responsible for the program.

It is recommended, while not prohibitive, that patients perform an exercise stress test (EST) and/or a cardiopulmonary exercise test (CPET) before starting their exercise program to identify the target training zone, just as recommended in traditional CVR programs.^32 , 33^If it is not possible to perform an EST or CPET, the Talk Test^34^or 6-minute walk test^35^can help identify the appropriate exercise intensity to improve cardiorespiratory fitness.^16^

The professional responsible for prescribing and monitoring exercise sessions, whether a physical education professional or physiotherapist, must carry out a detailed anamnesis, in addition to tests to assess functional capacity and anthropometry, to complement the information on the patient’s general condition before the exercises. Reassessments after the intervention are recommended, mainly to provide the patient with information about their progress in the program. Therefore, it is believed that the patient’s motivation and the chances of changing their lifestyle can be improved.

Patients with ventricular arrhythmia and/or significant symptomatic myocardial ischemia during the stress test are considered to be at greater risk for physical training monitored remotely. In these cases, the risks and benefits must be assessed individually.^19^

Regarding patient eligibility and safety, special attention should be given to patients with severe heart failure, frailty, orthopedic or cognitive impairment, who live alone, or who feel uncomfortable using technology.^10^

A contingency protocol in case of serious adverse events, such as cardiorespiratory arrest, or even moderate to mild adverse events, must be described in the CTR program and must informed to the patient, family members, and caregivers. The following procedures should be included in the program:

1. Emergency contacts: provide patients with the contact numbers for the Mobile Emergency Care Service (SAMU, in Brazil) and the Fire Department. These numbers must be accessible and visible, making it easier for anyone to call the service in case of an emergency.
2. Guidance for effective communication: instruct patients about the appropriate way to communicate with emergency responders
  - The person calling must inform the responder that the case involves a person with a heart disease, specifying the type of heart disease diagnosed;
  - The person calling must also inform the responder that the case involves a person who is practicing supervised exercise as part of a rehabilitation program;
  - The symptoms should be accurately described, such as chest pain, shortness of breath, and dizziness, among others.
  - The address and the location where the patient will receive care must also be informed.
3. Caregiver training: provide caregivers with a comprehensive video lesson on cardiopulmonary resuscitation (CPR) to prepare them for potential cardiorespiratory arrest situations. Alternatively, when possible, this guidance can be provided through a face-to-face lecture, in a hybrid model.

This plan aims to ensure that patients and caregivers are adequately prepared to respond effectively in emergencies, promoting a safer environment during telerehabilitation practice.

### Prescribing physical exercise sessions in telerehabilitation

The content of a remotely supervised exercise program should be personalized based on each patient’s individual goals, preferences, and functional abilities, similar to a traditional CVR program. The patient’s previous experience and knowledge of the chosen modality can increase adherence, motivation, and safety. Hybrid CVR models, which combine exercise sessions in specialized centers with CTR, have been the subject of research and are gaining popularity with evolving evidence.^4 , 36^

Physical training should be prescribed according to the FITT model (frequency, intensity, time/duration, and type of exercise).^1 , 37^The frequency of aerobic exercises should be at least three days a week, preferably every day. Alternating physical exercise with physical activities carried out during leisure or commuting can be a good strategy for increasing the level of physical activity. For resistance exercises, a frequency of at least twice a week is recommended, with an emphasis on activities that improve the skills necessary for the effective and safe performance of daily life activities, focusing on natural and functional movements, such as pushing, pulling, squatting, lifting, and rotating. Free weights, elastic bands, chairs, sticks, balls, overballs, and other accessories can be used.

For intensity prescription, individualized guidance is recommended, combining different variables obtained during the CPET or the EST with an individual score of subjective perception of effort (Borg) or talk test. The basic recommendation is to consider a moderate or moderate to high-intensity domain, when possible (low-risk patients), adjusted to the individual characteristics of each person.^1^In patients who perform the CPET, the heart rate observed at the aerobic and anaerobic thresholds can be used as a reference, starting close to the first threshold and progressing to the HR between thresholds 1 and 2. In the EST, the intensity can vary from 60% to 80% of the maximum HR achieved in the test, progressing to up to 90% of the maximum HR. 50 to 70% of the reserve HR can also be used for patients without testing. Sessions can last from 30 to 60 minutes. Different types of physical exercises can be performed: resistance exercises, mobility exercises, flexibility exercises, using body weight, walking, and exercises on ergometers.

It is worth noting that, given the different protocols of the studies carried out to date, the optimal intensity for CTR is not known. The recommendations follow a pattern similar to those described in CVR programs carried out in traditional centers. This enables additional studies and protocol standardization so that we can have more robust information. Adherence to the physical exercise program and its long-term maintenance are thought to have a greater impact on health outcomes than the intensity of the exercises performed.

Remote physical exercise sessions must be prescribed and supervised by qualified and trained physical education professionals and physiotherapists, preferably with experience in CVR programs in specialized centers. Before starting a remote program, phase II patients should have a minimum experience of 4 face-to-face sessions in a specialized center. This hybrid approach aims to provide patients with a perception of the efforts, guide and correct movements, and direct the practice of remote sessions. At this time, the patient should receive guidance on using the subjective perception of effort scale,^38^which should be used in conjunction with heart rate monitors during exercise sessions.

Still, during phase II, online exercise sessions must take place in real-time, with bilateral communication between the healthcare professional and the patient. The patient should use a vital signs monitor, such as a heart rate monitor or an oximeter, be accompanied by another person, and have emergency contacts within reach in case of adverse events. Minimizing risks and prioritizing safety are emergency recommendations.

In phase III, sessions can be asynchronous through video classes or individualized guidance via specific exercise applications.

Currently, exercise prescription apps are available for use in weight training gyms or with body weight. The exercises can be performed at home or in gyms asynchronously. These new applications are simple to use and display explanatory videos with information about how to perform the exercise, the number of sets, repetitions, and loads. This technology provides the delivery of personalized or group exercise programs, and gained prominence during the COVID-19 pandemic, being increasingly used among different populations.

## Conclusion

Considering that Brazil is a country of continental dimensions, cardiac telerehabilitation appears as a relatively simple and effective solution to enable a greater offer of CVR, especially in areas lacking this kind of healthcare service. Digital platforms enable universal training for different services, with the possibility of creating coordinating centers and specialized consultants. Telerehabilitation should ideally be part of every structured Cardiovascular Rehabilitation service in Brazil.

## *Supplemental Materials

For additional information, please click here.
